# Two New *Rhizobiales* Species Isolated from Root Nodules of Common Sainfoin (Onobrychis viciifolia) Show Different Plant Colonization Strategies

**DOI:** 10.1128/spectrum.01099-22

**Published:** 2022-08-25

**Authors:** Samad Ashrafi, Nemanja Kuzmanović, Sascha Patz, Ulrike Lohwasser, Boyke Bunk, Cathrin Spröer, Maria Lorenz, Ahmed Elhady, Anja Frühling, Meina Neumann-Schaal, Susanne Verbarg, Matthias Becker, Torsten Thünen

**Affiliations:** a Julius Kühn Institute (JKI)-Federal Research Centre for Cultivated Plants, Institute for Epidemiology and Pathogen Diagnostics, Braunschweig, Germany; b Julius Kühn Institute (JKI)-Federal Research Centre for Cultivated Plants, Institute for Plant Protection in Horticulture and Forests, Braunschweig, Germany; c University of Tübingen, Institute for Bioinformatics and Medical Informatics, Algorithms in Bioinformatics, Tübingen, Germany; d Leibniz Institute of Plant Genetics and Crop Plant Research (IPK) Gatersleben, Genebank Department, Seeland, Germany; e Leibniz Institute German Collection of Microorganisms and Cell Cultures (DSMZ), Braunschweig, Germany; f Technische Universität Braunschweig, Braunschweig, Germany; g Julius Kühn Institute (JKI)-Federal Research Centre for Cultivated Plants, Institute for National and International Plant Health, Braunschweig, Germany; h Julius Kühn Institute (JKI)-Federal Research Centre for Cultivated Plants, Institute for Crop and Soil Science, Braunschweig, Germany; Huazhong Agricultural University

**Keywords:** *Mesorhizobium*, rhizobia, environmental microbiology, genomics, phylogenetic analysis

## Abstract

Root nodules of legume plants are primarily inhabited by rhizobial nitrogen-fixing bacteria. Here, we propose two new *Rhizobiales* species isolated from root nodules of common sainfoin (Onobrychis viciifolia), as shown by core-gene phylogeny, overall genome relatedness indices, and pan-genome analysis. Mesorhizobium onobrychidis sp. nov. actively induces nodules and achieves atmospheric nitrogen and carbon dioxide fixation. This species appears to be depleted in motility genes and is enriched in genes for direct effects on plant growth performance. Its genome reveals functional and plant growth-promoting signatures, like a large unique chromosomal genomic island with high density of symbiotic genetic traits. Onobrychidicola muellerharveyae gen. nov. sp. nov. is described as a type species of the new genus *Onobrychidicola* in *Rhizobiaceae*. This species comprises unique genetic features and plant growth-promoting traits (PGPTs), which strongly indicate its function in biotic stress reduction and motility. We applied a newly developed bioinformatics approach for *in silico* prediction of PGPTs (PGPT-Pred), which supports the different lifestyles of the two new species and the plant growth-promoting performance of M. onobrychidis in the greenhouse trial.

**IMPORTANCE** The intensive use of chemical fertilizers has a variety of negative effects on the environment. Increased utilization of biological nitrogen fixation (BNF) is one way to mitigate those negative impacts. In order to optimize BNF, suitable candidates for different legume species are required. Despite intensive search for new rhizobial bacteria associated with legumes, no new rhizobia have recently been identified from sainfoin (Onobrychis viciifolia). Here, we report on the discovery of two new rhizobial species associated with sainfoin, which are of high importance for the host and may help to increase sustainability in agricultural practices. We employed the combination of *in silico* prediction and *in planta* experiments, which is an effective way to detect promising plant growth-promoting bacteria.

## INTRODUCTION

Rhizobia is a common term referring to a paraphyletic group of bacteria which are able to induce nodules on the roots of legumes and fix atmospheric nitrogen (N_2_). They have been investigated since the identification of their roles in nitrogen acquisition for legume plants (1, 2). Rhizobia show variability in their nodulation strategies. Some of them are host specific, while others can nodulate various plant species, even members of nonlegume plants (3). Rhizobia comprise a genetically diverse group of bacteria. They share a symbiotic nitrogen fixation function that is encoded on symbiotic plasmids or symbiosis islands within the genome (4, 5), jointly termed symbiotic genome compartments (SGCs) (6). Legume root nodules are principally inhabited by nitrogen-fixing bacteria. However, this ecological niche contains many other nonrhizobial bacterial species, collectively called nodule-associated bacteria (7, 8). They are involved in different biological activitie*s*, e.g., plant growth promotion and biocontrol (9). Nevertheless, our knowledge about the entire biological functions of nodule-associated bacteria is elusive.

Based on the current taxonomical information, rhizobia are classified within a number of families of the alphaproteobacterial order *Rhizobiales.* Non-nitrogen-fixing *Rhizobiaceae* members were also recovered from legume root nodules (10–13). Members of well-known rhizobial genera *Bradyrhizobium* (14) and *Mesorhizobium* (15) were initially classified into the genus *Rhizobium* but were later reclassified into separate genera and subsequently placed into the new families *Bradyrhizobiaceae* (16) and *Phyllobacteriaceae* (17).

Onobrychis viciifolia Scop. (Fabaceae), commonly referred to as common sainfoin, is an autochthonous leguminous plant with a putative origin in Central Asia. It was introduced to Europe in the 14th century and was intensively cultivated until the “green revolution,” during which it was replaced by higher-yielding legumes such as alfalfa (Medicago sativa). Onobrychis viciifolia is known as “healthy hay” (from its old French name, “Sain foin”) due to its positive effects on animal health and animal feeding (18–21). Despite these positive traits, sainfoin is lacking a widespread application in agriculture in northern Europe. One reason may be the reports of inadequate levels of nitrogen fixation, resulting in nitrogen deficiency symptoms despite the use of bacterial inocula (22–24). Although sainfoin has been shown to reach similar rates of nitrogen fixation (130 to 160 kg/ha) as alfalfa (140 to 160 kg/ha) (25), the rate is highly dependent on the efficiency of the associated rhizobial symbiont (26). Several rhizobia isolated from other legumes, including *Coronilla* spp., *Hedysarum* spp., *Petalostemon* spp., *Oxytropis* spp., and Astragalus alpinus, can also nodulate O. viciifolia (26, 27). However, not many studies reported rhizobial strains nodulating sainfoin (28).

In rhizobia, nitrogenase genes are part of symbiotic genome compartments (SGCs). Such large DNA fragments can be shared among bacteria by horizontal gene transfer via plasmids, integrative conjugative elements (ICEs), and/or genomic islands (GEIs) located on chromosomes. Andrews et al. (29) showed that symbiosis genes have been horizontally transferred within and between rhizobial genera. According to their gene content, GEIs and ICEs can be described as pathogenicity, symbiosis, metabolic, fitness, or resistance islands (6, 30–32). Both pathogenic genome compartments (pathogenicity islands, virulence plasmids) and symbiotic genome compartments (symbiosis islands, symbiotic plasmids) convert environmental strains to strains that are able to form close pathogenic or symbiotic associations with eukaryotic hosts (33). The community of rhizobial- and nodule-associated bacteria is assumed to exchange plant-beneficial traits by transferring SGCs. As an example, Sullivan and coworkers found that the transfer of the symbiosis island of Mesorhizobium loti strain ICMP3153 (derivative R7A) converted nonsymbiotic *Mesorhizobium* into plant symbionts (34).

In an attempt to identify rhizobial strains associated with sainfoin, different sainfoin varieties planted in an experimental field in the Leibniz Institute of Plant Genetics and Crop Plant Research (IPK), Germany, were screened. In this context, two new strains were isolated from root nodules of sainfoin plants. We investigated these strains to (i) characterize them using *in silico* and *in vivo* studies, (ii) elucidate their taxonomic affiliation, plant growth-promoting traits repertoire, and (iii) evaluate their plant-beneficial potential using greenhouse experiments.

## RESULTS

### Phylogenetic inferences.

A phylogenetic analysis based on a partial sequence of the 16S rRNA gene showed that the isolate OM4 formed a highly supported monophyletic group with strains Mesorhizobium delmotii STM4623^T^, Mesorhizobium prunaredense STM4891^T^, Mesorhizobium wenxiniae WYCCWR 10195^T^, Mesorhizobium muleiense CCBAU 83963^T^, Mesorhizobium robiniae CCNWYC115^T^, Mesorhizobium temperatum SDW018^T^, and Mesorhizobium mediterraneum NBRC 102497^T^ (see Fig. S1 in the supplemental material). Analyses of the housekeeping genes *recA* and *atpD* revealed a close relationship between OM4 and M. prunaredense STM4891^T^ with high branch support (Fig. S2A and B). In addition, whole-genome sequence analysis demonstrated a distant relationship between these strains (see below).

The 16S rRNA gene sequence comparison of isolate TH2 with related *Rhizobiaceae* members suggested a close relationship with Rhizobium alvei strain TNR-22^T^ (GenBank accession no. HE649224), sharing 98.08% nucleotide identity for an alignment length of 1,405 bp. This was below the stringent cutoff of 98.7% 16S rRNA sequence identity and was proposed to delineate new species (35). These two strains shared only 86.14% and 87.65% nucleotide identity for their partial *atpD* and *recA* gene sequences, respectively, suggesting their distinctiveness. The latter comparison was limited to 496- and 567-bp sequence lengths because only partial *atpD* (GenBank accession no. KX938336) and *recA* (GenBank accession no. KX938338) sequences for R. alvei TNR-22^T^ were available. The 16S rRNA and *recA*-based phylogenetic analyses demonstrated that the isolate TH2 and R. alvei formed a monophyletic group with high support values (Fig. S3; Fig. S4A). The *atpD*-based analysis resulted in a tree with different topology where TH2 did not cluster with R. alvei but with other representatives of *Rhizobium*, *Agrobacterium*, and *Ciceribacter* (Fig. S4B). Whole-genome analysis, however, showed a distant relationship between these strains (see below).

### Core genome phylogeny, overall genome relatedness indices, and plasmid comparison.

Core genome phylogeny was determined for isolates OM4 and TH2 and 99 additional *Rhizobiales* strains, including representatives of *Rhizobiaceae* and *Phyllobacteriaceae*. The core genome of strains included in this analysis contained 180 homologous gene clusters. A phylogenomic tree was inferred from 118 top markers that were selected using GET_PHYLOMARKERS software.

**FIG 1:**
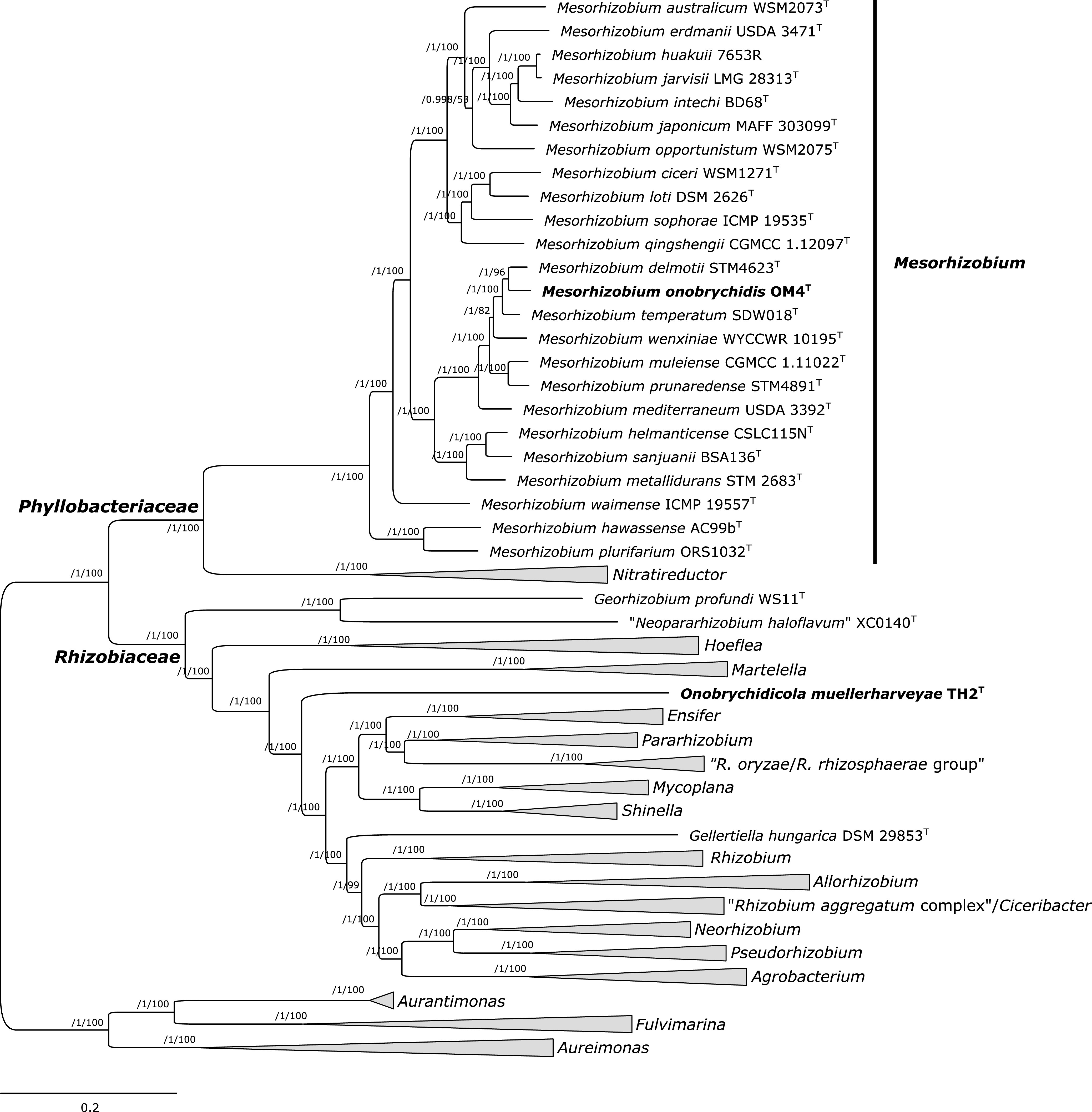
Maximum-likelihood core genome phylogeny of strains Onobrychidicola muellerharveyae TH2^T^ and Mesorhizobium onobrychidis OM4^T^, including representatives of *Rhizobiaceae* and *Phyllobacteriaceae* (genera *Mesorhizobium* and *Nitratireductor*). The tree was estimated with IQ-TREE from the concatenated alignment of 118 top-ranked genes selected using GET_PHYLOMARKERS software. The numbers on the nodes indicate the approximate Bayesian posterior probabilities support values (first value) and ultrafast bootstrap values (second value) as implemented in IQ-TREE. The tree was rooted using the sequences of representatives of genera *Aurantimonas*, *Aureimonas*, and *Fulvimarina* as outgroup. The scale bar represents the number of expected substitutions per site under the best-fitting GTR+F+ASC+R8 model. The same tree, but without collapsing clades (thick gray branches), is presented as Fig. S7 in the supplemental material.

The maximum-likelihood (ML) core genome phylogeny indicated that the isolate OM4 grouped within the genus *Mesorhizobium* (Fig. 1). It clustered with strains M. delmotii STM4623^T^ and M. temperatum SDW018^T^ as its closest relatives. Isolate OM4 exhibited the highest genomic relatedness to these two strains, as they shared ~94.8% average nucleotide identity based on BLAST (ANIb; Table S3). This was below the proposed threshold for species delineation, which ranges between 95 and 96% for ANI (36). To clarify the taxonomic assignment of the isolate OM4, we calculated additional overall genome relatedness indices (OGRIs), in particular, orthoANIu and digital DNA-DNA hybridization (dDDH). Obtained values were also below the thresholds for species definition (Table S3). This suggests that isolate OM4 represents a novel *Mesorhizobium* species for which we proposed the name Mesorhizobium onobrychidis sp. nov. (see Appendix). The novelty of M. onobrychidis strain OM4^T^ was also confirmed by Type (Strain) Genome Server (TYGS) analysis, suggesting that this strain does not belong to any species found in the TYGS database (data not shown).

Phylogenetic analysis assigned isolate TH2 to *Rhizobiaceae* (Fig. 1). It clustered independently and was distantly related to other *Rhizobiaceae* genera described so far. Different OGRIs were computed to further assess the relationship of isolate TH2 to representatives of *Rhizobiaceae*. Because of the distinctiveness of this isolate, the comparisons at the nucleotide level were not satisfactory, and only a limited proportion of the whole-genome DNA sequence could be used for the calculations. For instance, for ANIb, only ~12 to 26% of the whole-genome sequences were aligned and used for comparisons (data not shown). Therefore, we performed whole-proteome comparisons (whole-proteome average amino acid identity [wpAAI]) that offer a higher resolution. Isolate TH2 exhibited wpAAI values ranging from 61.5 to 67.5% with the representatives of *Rhizobiaceae* included in the analysis (Table S2). The wpAAI values were notably low and supported the divergence of the isolate TH2, which was evidenced by the separate clustering of the strain on a wpAAI dendrogram (Fig. S5). Isolate TH2 exhibited the highest genomic relatedness to strain Ensifer meliloti 1021 (67.5% wpAAI), although they were phylogenetically distantly related (Fig. 1; Table S3). This value was lower than wpAAI values computed between representatives of defined and phylogenetically well-separated genera *Agrobacterium* and *Rhizobium* that ranged from 68.12 to 70.55% wpAAI. The core-proteome average amino acid identity (cpAAI) between the isolate TH2 and reference *Rhizobiaceae* spp. was <76% (Table S4), which was below the threshold of ~86% for delimitation of *Rhizobiaceae* genera proposed recently (37). This suggested that isolate TH2 represents a new genus and species, described here as Onobrychidicola muellerharveyae gen. nov. sp. nov. The separate taxonomic position of strain O. muellerharveyae TH2^T^ was also confirmed by results of TYGS analysis (data not shown).

**FIG 2:**
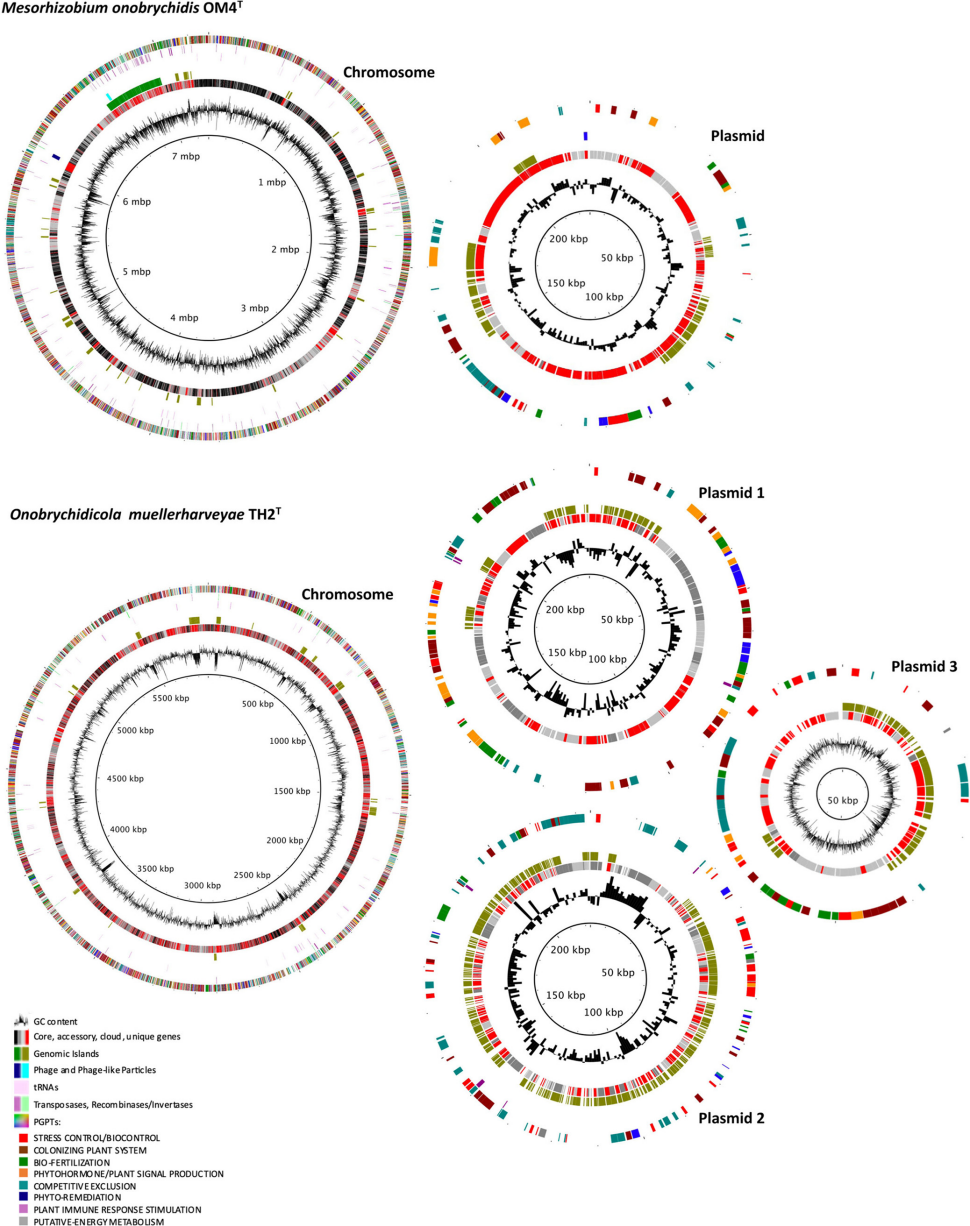
Genome annotation of Mesorhizobium onobrychidis OM4^T^ and Onobrychidicola muellerharveyae TH2^T^. Each chromosome and plasmid, respectively, are presented by a circular plot containing seven levels, of which the innermost circle 1 displays the G+C content of DNA. Circle 2 summarizes the Roary core genome results with highlighted core (black), accessory (dark gray), cloud (light gray), and strain-specific (unique) genes (red). Circle 3 presents distribution of genomic island genes predicted by IslandViewer version 4. Among the remaining circles, circle 4 demonstrates the genes encoding phages or phage taillike particles, circle 5 shows tRNAs, and circle 6 shows transposases (violet) or recombinases/invertases (turquoise), i.e., enzymes enabling genome reshuffling. The outermost circle 7 presents genes annotated to plant growth-promoting traits (PGPTs) by PGPT-Pred, here subdivided into eight functional classes on PGPT ontology level 2.

Plasmids of M. onobrychidis OM4^T^ and O. muellerharveyae TH2^T^ did not show high similarity to known plasmids based on mash analysis and pan-genome analysis and revealed a high proportion of unique genes (Fig. 2, Fig. S6, and Text S7).

### Genome sequencing and assembly.

Genomes of strains M. onobrychidis OM4^T^ and O. muellerharveyae TH2^T^ were sequenced and circularized employing PacBio and Illumina platforms upon completion. Basic genome assembly statistics of both strains are summarized in Table 1.

**TABLE 1:** Genome statistics of Mesorhizobium onobrychidis OM4^T^ and Onobrychidicola muellerharveyae TH2^T^^*a*^

Characteristic	Data for:	
	Mesorhizobium onobrychidis OM4^T^	Onobrychidicola muellerharveyae TH2^T^
Genome content	Chromosome and one plasmid	Chromosome and three plasmids
Genome size	Total, 7.55 Mb (C, 7.32 Mb; P1, 227 kb)	Total, 6.44 Mb (C, 5.88 Mb; P1, 238 kb; P2, 223 kb; P3, 98 kb)
GC content (%)	C, 61.94; P1, 59.8	C, 60.64; P1, 59.75; P2, 57.55; P3, 56.31
No. of genes	7,415 (7,347 CDS)	6,373 (6,312 CDS)
No. of hypothetical genes	3,779	3,157
KEGG-annotated genes	3,676	3,365
No. of unique genes (PG)	1,068	2,261
No. of unique hypothetical genes (PG)	847	1,729
No. of rRNA operons (5S, 23S, 16S)	2	2
No. of tRNAs	62	53
No. of phages, PLPs, and transposases	2 PLPs; 136 transposases	1 Phage; 3 PLPs; 18 transposases
No. of recombinases/invertases (e.g., *xerC*, *xerD*)	27	13
No. of PGPTs (density [0,1])	C, 3,973 (0.5545); P, 65 (0.3591)	C, 3,270 (0.57); P1, 110 (0.5263); P2, 227 (0.3910); P3, 50 (0.50)
SI or SP (density [0,1]) with GC content (%)	SI 1, 421 kb (0.7542); GC, 59.51	

a PG, pan-genome; PLPs, phage-like particles; SI, symbiosis island; SP, symbiosis plasmid; C, chromosome; P1, plasmid 1; P2, plasmid 2; P3, plasmid 3.

For strains OM4^T^ and TH2^T^, 80,208 and 80,011 postfiltered long reads with mean polymerase read lengths of 14,639 and 14,004 bp have been generated, respectively. Long-read assembly with a target genome size of 15 Mb resulted in 6 (OM4^T^) and 2 (TH2^T^) contigs, respectively, with 4 (OM4^T^) and 2 (TH2^T^) of them being circular and, as such, handled as final replicons. For short-read error correction, a total of 2 × 1,572,218 (OM4^T^) and 2 × 2,942,298 (TH2^T^) reads of 151 bp have been trimmed and mapped to the complete genome for error correction. No contamination has been detected based on the obtained replicons. Genome completeness has been evaluated using BUSCO (https://usegalaxy.eu/) by manual selection of the *Rhizobiales* lineage as reference (639 BUSCOs). Here, in both cases, 638 complete BUSCOs were detected, reaching completeness values of 99.9%.

The complete genome size of strain M. onobrychidis OM4^T^ was 7.55 Mb, comprising the circular chromosome of 7.32 Mb and one circular plasmid of 227 kb, and is stored in the NCBI GenBank under accession numbers CP062229 and CP062230 (Fig. 2). The G+C content of the total genome is 61.9%. Genome size and G+C content of strain M. onobrychidis OM4^T^ are similar to other *Mesorhizobium* spp. (Table S5), for instance, the type strain of this genus, strain Mesorhizobium loti DSM 2626^T^ (GenBank accession no. QGGH01000000).

The genome of strain O. muellerharveyae TH2^T^, composed of the circular chromosome (5.88 Mb) and three circular plasmids (98 kb, 223 kb, and 238 kb), was deposited under the accession numbers CP062231 to CP062234 at NCBI GenBank (Fig. 2). The genome size and G+C content of the total genome were 6.44 Mb and 60.6%, respectively, which was similar to other representatives of *Rhizobiaceae* (Table S5).

Two chromosomal rRNA (5S, 23S, and 16S) operons were identified in both strains OM4^T^ and TH2^T^. In O. muellerharveyae TH2^T^, they were identical, while rRNA operons of strain M. onobrychidis OM4^T^ differed in five single nucleotide polymorphisms (SNPs) located in the intergenic region. For M. onobrychidis OM4^T^, two phage-like particles (PLPs) were identified, while in O. muellerharveyae TH2^T^, one phage and three PLPs were found (Fig. 2, circle 4). Mesorhizobium onobrychidis OM4^T^ harbored 136 transposases and 33 recombinases/invertases, e.g., *xerC* and *xerD*, whereas O. muellerharveyae TH2^T^ revealed respective counts of 28 and 13 only (Fig. 2, circle 6). Approximately half of the genes of both genomes were lacking meaningful annotations (hypothetical genes) according to homology-based alignment by PROKKA and KOfamKOALA hidden Markov model (HMM) searches. According to the genomic island prediction tool IslandViewer 4, M. onobrychidis OM4^T^, but not O. muellerharveyae TH2^T^, contains a very large genomic island on its chromosome, harboring 414 genes (Fig. 2, circle 3; Fig. S8A). This is the only larger fragment of the M. onobrychidis OM4^T^ chromosome with a high density of unique genes (Table S1). The genomic island on the M. onobrychidis OM4^T^ chromosome is located next to unique genes that are enriched in particular functions such as catalyzing DNA exchange. The plant growth-promoting trait (PGPT) density of the genomic island is 75%, considerably higher than the average PGPT density of the entire chromosome, which is only 55% (see also Table 1). Details regarding the differences of PGPTs between M. onobrychidis OM4^T^, O. muellerharveyae TH2^T^, and other closely related strains are provided below.

### Comparative genomics and functional annotation.

Pan-genome analysis of strains M. onobrychidis OM4^T^ and O. muellerharveyae TH2^T^ revealed a large number of gene clusters, ranging from 36,631 for all *Mesorhizobium* strains to 85,606 for all *Rhizobiaceae* strains here analyzed (Fig. 2, circle 2; Fig. S9A and B). The strain M. onobrychidis OM4^T^ revealed 2,683 core, 1,151 accessory, 2,444 cloud, and 1,068 unique genes. While 428 unique genes could not be assigned to any KO number (KEGG annotations), 441 KO numbers were detected for M. onobrychidis OM4^T^ as unique genes, with various gene copy numbers. Functions of unique genes were associated with, among others, prokaryotic cellular community, signal transduction, carbohydrate and amino acid metabolism, cofactor and vitamin biosynthesis, energy metabolism, membrane transport, and lipid metabolism (Fig. S10). In contrast, the putative novel genus comprising single strain O. muellerharveyae TH2^T^ revealed only 1,107 core genes, while counts of 1,839 for accessory, 1,105 for cloud, and 2,261 for unique genes were scored. The results did not allow further pan-genomic analysis for O. muellerharveyae TH2^T^ due to a distant phylogenetic relation between O. muellerharveyae TH2^T^ and the strains here analyzed.

Overall, KEGG functional analysis and respective abundance clustering of all KEGG annotations confirmed that M. onobrychidis OM4^T^ contained functional similarities to M. delmotii STM4623^T^ and M. temperatum SDW018^T^. The analysis also supported the novelty of this species when considering only strain-specific enriched K numbers (Fig. S9C). Analyzing all K numbers for O. muellerharveyae TH2^T^ resulted in a distinct clustering, which became more distinct when considering only enriched ones (Fig. S9D). Both patterns highly supported its status as a new genus.

The KEGG functional annotation for M. onobrychidis OM4^T^ and O. muellerharveyae TH2^T^ revealed two distinct clusters of level 2 and level 3 KEGG functions (Fig. S11; Text S8). Onobrychidicola muellerharveyae TH2^T^ showed higher counts for genes related to membrane transport, cell motility, cell growth and death, antimicrobial drug resistance, signal transduction, and replication, repair, transport, and catabolism (Fig. S11A).

The detection of specific secondary metabolite biosynthesis gene clusters (BGCs) further confirmed the different lifestyles of strains OM4^T^ and TH2^T^ (Fig. S12; Text S9). The whole-genome alignment of *Mesorhizobium* spp. revealed 85 regions unique to M. onobrychidis OM4^T^, harboring at least 5 and up to 77 genes as one of its novel species characteristics (Fig. S13, Table S1; Text S10). Twenty-one regions could be assigned to 7 of the entire 11 BGCs of OM4^T^. Among them, 2 BGCs matched with the genomic island, which covers 63 unaligned regions, including 364 genes, all assigned as unique genes (Fig. S14; Table S2; Text S10).

### Functional PGPT annotation.

**FIG 3:**
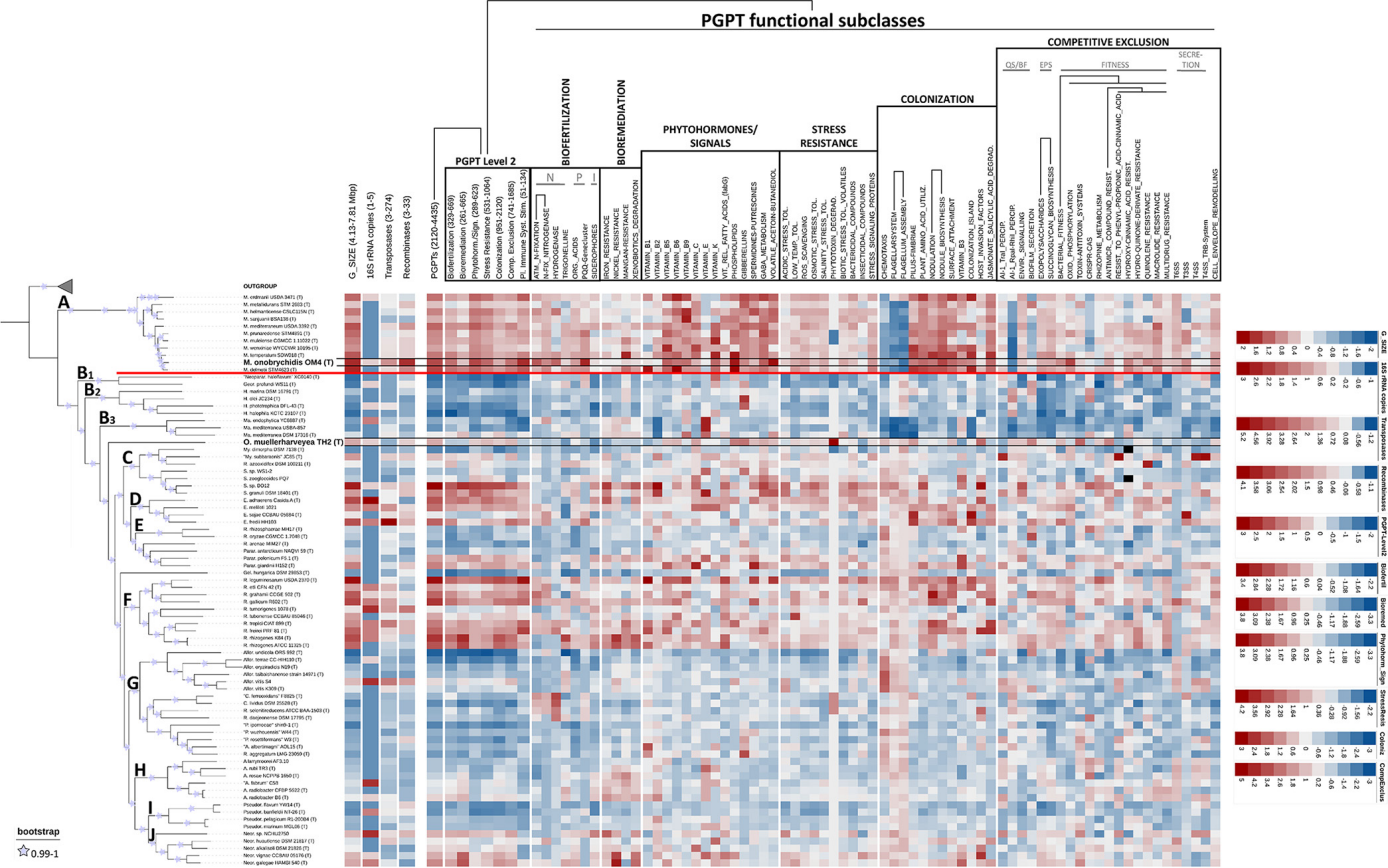
Functional PGPT heatmap based on KEGG annotations highlighting PGPT abundant differences in functional classes and important genetic characteristics of Mesorhizobium onobrychidis OM4^T^, Onobrychidicola muellerharveyae TH2^T^, and other *Rhizobiaceae* and *Phyllobacteriaceae*. The reddish color shows enriched, and bluish color shows depleted gene numbers based on a trait-specific z-scale, which is given on the right. The phylogenetic tree provided on the left-hand side allows better understanding of the PGPT distributions. Respective phylogenetic clades are highlighted by capital letters within the tree.

The main genetic features and functional PGPT annotations, based on KOfam-KEGG for PGPT mapping of all 80 strains, are summarized in a heatmap (Fig. 3). Detailed values are given in Table S1. The pattern of depleted (blue) and enriched (red) traits coincided with the phylogenetic clades apart from very few exceptions in clade C. Heatmap fractions belonging to the *Mesorhizobium* clade (clade A), *Ensifer* clade (clade D), and *Rhizobium* clade (clade F) were dominated by PGPT classes of enriched gene counts.

Focusing on the *Mesorhizobium* clade, a fraction of depleted traits refers to three subclasses of the PGPT class “colonization,” namely, chemotaxis, flagellar system, and flagellum assembly, important for bacteria to migrate toward chemical stimuli. In total, 4,046 genes of M. onobrychidis OM4^T^ could be allocated to PGPTs, compared to an average of 3,735 genes among other *Mesorhizobium* strains. A similar PGPT count was also found for its closest relative, M. delmotii STM4623^T^. In general, the newly described species, M. onobrychidis, is very similar to the other species of the genus *Mesorhizobium*. Among *Mesorhizobium*, M. onobrychidis OM4^T^ is one of the strains with the highest number of genes in the following PGP level 2 classes: biofertilization, phytohormone, plant signal production, stress resistance, competitive exclusion, and plant immune response stimulation. PGPT counts of M. onobrychidis OM4^T^ for the mentioned phytohormone and plant signals and plant immune system stimulation traits were higher than its two closest relatives, M. delmotii STM4623^T^ and/or M. temperatum SDW018^T^ (Table S1). In contrast, M. onobrychidis strain OM4^T^ showed only an average amount of bioremediation genes, distinguishing it merely from M. delmotii STM4623^T^ and M. temperatum SDW018^T^. Mesorhizobium onobrychidis OM4^T^ harbored genes related to fixing carbon dioxide via ribulose 1,5-bisphosphate carboxylase/oxidase (Rubisco) as another highly plant-beneficial feature (data not shown). It comprised a versatile set of stress resistance and colonization genes; their abundance mostly coincided with its both closest relatives. Furthermore, it contained the genetic ability for nodulation, vitamin B_3_ and pilus-fimbriae biosynthesis, the use of plant-derived metabolite*s*, e.g., amino acids, and the degradation of jasmonate/salicylic acid. Traits related to competitive exclusion showed a higher PGPT count for bacterial fitness than all other *Mesorhizobium* strains, especially for oxidative phosphorylation and resistance against plant antimicrobial compounds hydroxycinnamic acid and quinoline. The most significant differences between M. onobrychidis OM4^T^ and other *Mesorhizobium* strains occurred in the number of transposases and *xerC*/*xerD* recombinases, which are important PGPTs related to colonization and competitive exclusion. Mesorhizobium onobrychidis OM4^T^ has approximately 2.5 times as many genes belonging to these categories as the other *Mesorhizobium* strains on average (transposases, 136 compared to 57; recombinases, 33 compared to 13). Regarding secretion systems, M. onobrychidis OM4^T^ encoded one type VI secretion system (T6SS), two T3SSs, and one T4SS (*trb*) on its chromosome, as well as one copy of the *virB*-specific T4SS on its plasmid. The PGPT distribution alternated in a shared pattern or highly varied between M. onobrychidis OM4^T^ and its relatives M. delmotii STM4623^T^ and/or M. temperatum SDW018^T^.

Onobrychidicola muellerharveyae TH2^T^ strongly differed in its overall PGPT abundancy profile from any other phylogenomic clades (Fig. 3, B1 to B3 and C to J). It contained a rather low number of genes for biofertilization and bioremediation, while the classes phytohormone and plant signaling, stress resistance, colonization, and competitive exclusion were slightly above average. Onobrychidicola muellerharveyae TH2^T^ is one of the *Rhizobiaceae* strains with the highest phospholipid- and gibberellin-encoding PGPT count. In terms of stress resistance, O. muellerharveyae TH2^T^ exceeds all other strains in the copy number of the gene for tabtoxin degradation (*ttr*), which is produced by some plant pathogens. While most *Rhizobiaceae* have one tabtoxin degradation gene (Fig. 3), O. muellerharveyae TH2^T^ contained four copies of this gene. In terms of competitive exclusion, O. muellerharveyae TH2^T^ was superior to all other investigated strains concerning the enrichment of genes for toxin-antitoxin systems (TASs). This is the case also in biofilm secretion and resistance to antimicrobial compounds. In terms of (host) colonization, O. muellerharveyae TH2^T^ was remarkable in the subclass “host invasion factors” and subclasses that enable target-oriented movement (chemotaxis, flagellar system, and flagellum assembly). However, it lacked the nodulation gene cluster despite possessing single nodulation-associated genes like *nolA* and *nodD*. It showed an exceptional higher gene count for plant-branching inhibition and embryogenesis compounds spermidine and putrescine that act as plant signals. Regarding secretion systems, O. muellerharveyae TH2^T^ only harbored one T4SS (*virB*) on plasmid 2 and one T2SS on plasmid 3.

### Phenotypic characterization and fatty acid analysis.

Phenotypic characteristics of strains O. muellerharveyae TH2^T^ and M. onobrychidis OM4^T^ are summarized in Table S6. Differential characteristics of O. muellerharveyae TH2^T^ and the type species from the other genera of the family *Rhizobiaceae* are indicated in Table S5. Phenotypic tests performed with the API 20NE system and Biolog GEN III microplates were assessed as unreliable since negative reactions were observed for the majority of tests (data not shown). This was likely because of the growth conditions that were inadequate for these strains. Therefore, most of the tests included in the API 20NE system were repeated as conventional biochemical assays in test tubes in order to facilitate monitoring of bacterial growth and result assessment. Although more satisfactory results were obtained this way, no bacterial growth was observed for some tests, i.e., in media containing l-tryptophane as a substrate (indole production test). For strain M. onobrychidis OM4^T^, no bacterial growth was observed in media containing esculin-ferric citrate (esculin activity test) and gelatin (esculin hydrolysis test) as the substrates.

The results of the fatty acid analysis are summarized in Table S7. The major fatty acids (>5%) of O. muellerharveyae TH2^T^ are C_18:1_ ω7c, C_19:0_ cyclo ω7c, and C_16:0_ and C_17:0_ cyclo ω7c. Generally, as in other *Rhizobiaceae* members, the dominant fatty acid in O. muellerharveyae TH2^T^ was C_18:1_ ω7c, which is, in some strains, comprised in summed feature 8 (C_18:1_ ω7c/C_18:1_ ω6c). Unlike other type species from the other genera of *Rhizobiaceae*, O. muellerharveyae TH2^T^ contained a relatively high (>5%) amount of C_17:0_ cyclo ω7c. For M. onobrychidis OM4^T^, the major fatty acids (>5%) were C_18:1_ ω7c, C_16:0_, C_19:0_ cyclo ω7c, and 11 methyl C_18:1_ ω7c and C_18:0_, similar to other *Mesorhizobium* spp. (38).

### Plant nodulation and growth experiment.

**FIG 4:**
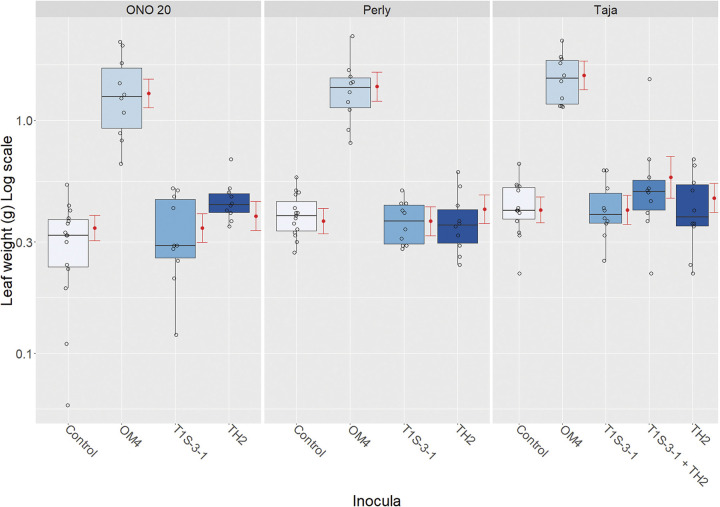
Plant biomass of nodulation assay using sainfoin accession ONO 20 and varieties Perly and Taja inoculated with Mesorhizobium onobrychidis OM4^T^ or Onobrychidicola muellerharveyae TH2^T^. Rhizobium leguminosarum T1S-3-1, known to induce nodulation, was used as a positive control. Onobrychidicola muellerharveyae TH2^T^ and R. leguminosarum T1S-3-1 were inoculated together into variety Taja as an attempt to piggyback O. muellerharveyae TH2^T^ into sainfoin plants. The negative control (“Control”) received no inoculation. Red dots and error bars show results of GLM statistical analysis.

Nodulation and plant growth promotion assays confirmed Koch’s postulates for strains M. onobrychidis OM4^T^ and the control Rhizobium leguminosarum TS1-3-1. Both strains could be reisolated from surface-sterilized nodules. Reisolation of O. muellerharveyae TH2^T^ failed for both single inoculations and coinoculation with R. leguminosarum TS1-3-1. Sainfoin inoculated with M. onobrychidis OM4^T^ showed a statistically significant gain in aboveground biomass of all three tested sainfoin varieties (Fig. 4). Plants treated with O. muellerharveyae TH2^T^ did not exhibit increased biomass.

## DISCUSSION

### Bioinoculant potential and host interaction.

The functional KEGG analyses revealed two contrasting settings for M. onobrychidis OM4^T^ and O. muellerharveyae TH2^T^, indicating two different lifestyles. Both strains differ in their competitive strategies, especially for colonizing the plant system. *In silico* analysis of PGPTs highlights the potential of M. onobrychidis OM4^T^ to improve plant performance via biofertilization, phytohormone, and plant signal production. Genes for root colonization and adhesion by nodulation (*nod* gene cluster) and biotin metabolism were highly enriched in M. onobrychidis OM4^T^, whereas O. muellerharveyae TH2^T^ revealed a higher count for genes affiliated with motility, chemotaxis, and host invasion.

Onobrychidicola muellerharveyae TH2^T^ possessed all genes to assemble a complete flagellum with three different copies of the flagellin gene (*fliC*) allowing presumably higher diversity of flagellin epitopes acting as microbe-associated molecular patterns (MAMPs). Allelic variation of *fliC* is employed by bacteria to avoid the plant immune response (39). Onobrychidicola muellerharveyae TH2^T^ lacked the nodulation gene cluster but harbored two nodulation-associated genes (*nolA* and *nodD*). Assuming that O. muellerharveyae TH2^T^ is not capable of inducing nodulation, this strain can be considered only a nodule-associated strain. Our greenhouse experiments confirmed the *in silico* analysis.

Although it carries a large amount of PGP genes, O. muellerharveyae TH2^T^ showed no effect on sainfoin plants in our inoculation experiment under nitrogen-limited conditions. Onobrychidicola muellerharveyae TH2^T^ might achieve better potential under biotic stress conditions (9), as its highest number of genes were found in functional classes referring to bacterial fitness/stress tolerance. In contrast to most other *Rhizobiaceae*, which have only one copy, O. muellerharveyae TH2^T^ had four copies of the tabtoxin degradation gene (*ttr*). Plant pathogens such as Pseudomonas syringae produce tabtoxin for chlorosis and lesion formation (40) and carry a *ttr* gene for self-protection from tabtoxinine beta-lactam (41). It can be assumed that the *ttr* gene products of O. muellerharveyae TH2^T^ diminish the deleterious effect of phytotoxin-producing bacteria.

Among all analyzed bacteria, O. muellerharveyae TH2^T^ contained the highest fraction of genes belonging to toxin-antitoxin systems (TASs). It is uncertain whether TAS provides any advantage to its host plant since plant-pathogenic bacteria such as Xylella fastidiosa also employ TASs (42). It has been argued that TASs do not necessarily provide an advantage to the producing bacterial strain. For example, chromosomal TASs of Pseudomonas putida were reported to be rather selfish than beneficial, and an indirect positive effect for plants cannot be ruled out (43). This example on TASs illustrates the need for further functional studies of particular genes and shows the difficulty of assigning them to a unique purpose.

Further effort is needed to identify plant-beneficial traits for robust and reliable prediction of PGPTs. However, M. onobrychidis OM4^T^ provides a convincing example that *in silico* analyses can already be used for the identification of bacterial strains exhibiting a beneficial impact on plants. Here, we showed that comparative genomics of PGPTs, based on novel ontology, is a solid tool that considers widely acknowledged PGP pathways such as nitrogen and carbon dioxide fixation together as one entity (biofertilization). The use of bioinformatics for determining genomic islands, e.g., via IslandViewer combined with PGPT enrichment analyses, demands a reclassification of symbiotic island and symbiotic plasmids, as not all criteria defined by Ling et al. (44) match. The lack of these genes in the genus *Mesorhizobium* (exception for strain USDA 3471) and in M. onobrychidis OM4^T^ suggests that most of the *Mesorhizobium* strains included in our *in silico* analyses are immotile or at least do not move by means of flagella.

### Genetic features of OM4.

Mesorhizobium onobrychidis OM4^T^ possessed 136 transposases (and 33 *xerC*/*xerD* recombinases), which is extraordinarily high among the representatives of *Phyllobacteriaceae* and *Rhizobiaceae* used in our analyses (Table 1; Fig. 3). A higher rate of transposable elements can be related to sessile endosymbiotic bacteria (45). However, this pattern was associated with reductive genome evolution of such sessile strains, which is not given for the strain described here. It has been discussed that the development of the nitrogen-fixing symbioses in legume nodules required coevolution of legumes and rhizobia (46). Zhao et al. (47), however, showed that adaptive evolution of symbiotic compatibility could be achieved by spontaneous transposition of inserted sequences (ISs). This was demonstrated by the observation that different *Sinorhizobium* strains do form either nitrogen-fixing nodules or uninfected pseudonodules (47). Next to ISs, site-specific recombinases *xerC* and *xerD* contribute to genome plasticity and mediate, e.g., formation and resolution of plasmid cointegrates (48). It was shown that *xerC* is crucial for competitive root colonization (49, 50). Accordingly, the high number of *xer*C/*xer*D genes in M. onobrychidis OM4^T^ suggests its competitive root colonization ability (49, 51).

Particular secretion systems are known to be crucial for bacteria-plant interaction (T1SS, T3SS, T4SS), competitive plant colonization (T6SS) (52), and plasmid transfer across the rhizobial community (T4SS) (53). Type III secretion systems (T3SSs) are well-known for effector translocation into eukaryotic host cells and are thus a major mediator for pathogenicity (54, 55). Such systems are, however, found to be present in symbiotic bacteria, where they contribute to a stable host-microbe interaction (56, 57). Mesorhizobium onobrychidis OM4^T^ encodes two T3SSs and two T4SSs, suggesting an effective interaction with its host.

One genomic region of M. onobrychidis OM4^T^ fulfilled some, but not all, of the criteria of a symbiosis island defined by Ling and coworkers (44). The tool IslandViewer, however, supported its nature as a genomic island. The presence of a higher density of PGPTs on this island compared to the density of the total genome raised the question of whether the criteria of a symbiosis island have to be extended by the PGPT density. PGPT annotation is challenging and not standardized. The use of the novel PLaBAse database and supporting online tools closes this gap (58).

Based on our functional analysis, the following functional characteristics for M. onobrychidis OM4^T^ can be proposed. It is a rather sessile strain, as it lacks the genes for chemotaxis and flagellar assembly. The strain is adapted to the plant metabolism, as it does not harbor an enriched set of carbohydrate, amino acid, and nucleotide metabolic genes compared to other plant-associated bacteria here analyzed. It carries a remarkable set of direct plant growth promotion traits and might achieve its colonization toward or inside the plant via biofilms and/or seed transmission (59).

The observed growth promotion during the greenhouse experiments suggests the bacterium as an “efficient” rhizobial species for sainfoin (O. viciifolia) under nitrogen-limited plant growth conditions.

### Conclusion.

The economic benefit of these newly discovered species still needs to be determined, but their phylogenetically distant position suggests them to be interesting research subjects. Onobrychidicola muellerharveyae TH2^T^ is the type strain of the monotypic genus *Onobrychidicola.* Since closely related strains have not been described, a large fraction of its genes is unique due to the overall low homology of genes. Onobrychidicola muellerharveyae TH2^T^ carries a high proportion of PGPTs that contribute to colonization, stress resistance, and competitiveness, rather than to direct plant-beneficial effects. Sufficient PGP potential for commercial application needs to be determined further in *in planta* experiments. Due to its likely potential to antagonize phytopathogens, the strain still could be considered for biocontrol purposes while developing alternatives to chemical pesticides.

A number of recent studies suggested sainfoin be integrated into modern and sustainable agriculture due to its beneficial properties on animal nutrition and animal and soil health (18). Overall performance of sainfoin highly depends on an effective symbiosis with rhizobial strains, many of which do not meet the plant’s nitrogen requirements (26). The study presented here describes the well-performing novel plant growth-promoting bacterial species M. onobrychidis OM4^T^, which is supported by its PGPTs. Our greenhouse experiments showed that this bacterium can be inoculated into a variety of sainfoin cultivars to improve their biomass production and might be a promising candidate for application in a sustainable agricultural system.

## MATERIALS AND METHODS

### Plant material sample collection.

One accession (ONO 20) and one cultivar (Taja) were selected for the present study. These plants were selected because of favorable characteristics like high tannin content and high biomass, respectively. ONO 20 is an old East German cultivar named Bendelebener D4 (60), which was included in GenBank in 1958. Taja is a registered cultivar from the Polish breeder Malopolska Hodowla Roslin Spolka z.o.o in Krakow. The plants were cultivated in experimental fields of the Leibniz Institute of Plant Genetics and Crop Plant Research (IPK) in Gatersleben, Germany, from 2017 to 2019. The fields contain loamy soil, are very fertile, and have high ground points (85 to 95). Content of organic matter is around 3%, pH value is 7.4, and nutrient concentration is 11.4 mg/100 g P, 21.3 mg/100 g K, and 12.8 mg/100 g Mg.

### Isolation of bacteria from root nodules.

Plant roots were washed to remove soil debris. Nodules were excised and their surfaces sterilized for 1 min in 70% ethanol, and they were rinsed twice with sterile deionized water (SDW), followed by incubation in 1% sodium hypochlorite (NaOCl) for 10 min and six rinses with SDW (61). Surface-sterilized nodules from each root sample were separately transferred to 2-mL microtubes and crushed with sterile pestles. Tubes were filled with 1 mL of SDW or sterile 10 mM MgCl_2_ and vortexed for 1 min. An 8-fold serial dilution was made from a 1-mL subsample of the homogenized suspension. A 100-μL subsample of each dilution was plated onto yeast mannitol agar (YMA; Sigma-Aldrich, Merck KGaA, Darmstadt, Germany) supplemented with Congo red. Plates were incubated at 28°C and monitored daily for 8 days. The bacterial strains, including isolates studied here (OM4 and TH2), were transferred to 2-mL cryogenic tubes filled with a mixture of yeast mannitol broth and 15% glycerin and stored at −80°C.

### DNA extraction, sequencing, and genome assembly.

For details regarding DNA extraction, amplification, and sequencing of partial 16S rRNA, *atpD*, and *recA* genes, as well as complete genome sequencing and assembly, see Text S1 in the supplemental material.

### Phylogenetic analysis.

Phylogenetic analysis was performed based on partial sequences of 16S rRNA gene and housekeeping genes *recA* and *atpD* and also a large number of conserved core genes. For more details, see Text S2.

### Overall genome relatedness indices.

For genus and species delimitation, we calculated various overall genome relatedness indices (OGRIs), including whole-proteome average amino acid identity (wpAAI) (62, 63, 64), core-proteome average amino acid identity (cpAAI), average nucleotide identity (ANI) (36, 62), and digital DNA-DNA hybridization (dDDH) (65). To further determine the taxonomic position of the isolates studied here (TH2 and OM4), their genome sequences were subjected to the Type (Strain) Genome Server (TYGS) pipeline for a whole genome-based taxonomic analysis (66). For more details, see Text S3.

### Plasmid similarity estimation.

Plasmid similarity to known plasmid sequences was calculated via Mash v.2.3 (67) in default dist mode. Respective reference plasmid sequences were received from the Plasmid database PLSDB version 2020_06_29 (68) and the Refseq plasmid collection stored at https://ftp.ncbi.nlm.nih.gov/genomes/refseq/plasmid/. For all plasmid reference hit sequences that showed at least one overlapping k-mer hash (Table S1), the pairwise mash distances were recalculated (sketch size, 10,000; k-mer size, 15) and visualized as neighbor network (NNet2004) by the outline algorithm with SplitsTree 5 v.5.2.4 (69, 70).

### Comparative genomics and whole-genome alignment.

A pan-genome analysis was performed for both isolates, TH2 and OM4, separately due to the different phylogenetic relationship, which was obtained by core genome phylogenomic analysis. Computation of a common pan-genome of both isolates failed due to high evolutional distance between both, which led to a significantly decreasing number of core genes. For best comparability during downstream analysis, all genomes were annotated with Prokka v.1.14.6 (71). Roary (72) v.3.13.0 (72) was applied to the annotated genomes of both isolates, using default parameters. The identity threshold (-i) was set differently according to the respective wpAAI values (Table S2) obtained for isolate TH2 (60%) and isolate OM4 (80%), considering the respective group gene/protein similarity. The single nucleotide polymorphism (SNP) tree of core genes was generated with FastTree v.2.1 (73) based on the maximum-likelihood method and the generalized time-reversible (GTR) model of evolution (parameters, -nt -gtr).

The genomes of isolate OM4 and its close relatives, M. delmotii STM4623^T^ and M. temperatum SDW018^T^, were aligned using MAUVE (snapshot_2015_02_25, default parameters) (74) to find OM4-specific genomic features. Isolate OM4-specific unaligned regions that did not belong to any locally colinear block (LCB; weight of 52) were extracted from the alignment file to analyze their functional characteristics and unique gene content.

### Genomic functional annotation and visualization.

Functional KEGG annotations were achieved for all isolates with the KOfamKOALA command line tool (https://www.genome.jp/tools/kofamkoala/) that applies HMM searches. KEGG comparisons between genomes were calculated and visualized with MEGAN6 (75) and custom Python scripts.

Genomic islands of the isolates TH2 and OM4 were detected online by IslandViewer 4 (76) using default parameters. Genomic prophage and phage-like regions were determined by the web tool PHASTER (77, 78). AntiSMASH v.6.0.1 (79) analysis allowed annotation of secondary metabolite biosynthesis gene clusters (BGCs). Selected annotation features were displayed as circular genome plots with BRIG (80). Unique genes of isolate OM4 were analyzed in more detail regarding their functional annotation and genomic position and affiliation to BGCs.

### Genes associated with plant-bacteria symbiosis and PGP.

The KEGG annotations of the proteins of all strains were parsed into an IMG-like KEGG annotation file format via an in-house script and mapped against the plant growth promotion traits ontology with the PGPT-Pred tool, available on the web platform for plant-associated bacteria, PLaBAse (58; http://plabase.informatik.uni-tuebingen.de/pb/plabase.php). The PGPT annotations of all strains were then merged for comparison. The PGPT density was calculated by division of the PGPT count by the total coding sequence (CDS) count of the respective genomic element (chromosome, plasmid, or genomic region). The PGPT count comparison was plotted as a z-scaled heatmap with iTol (81).

### Phenotypic characterization and fatty acid analysis.

For details regarding phenotypic characterization and fatty acid analysis, see Text S4 and S5.

### Plant growth promotion assays.

Reinoculation and nodulation tests were conducted as described in detail in Text S6.

### Data availability.

Genome sequences are available at NCBI GenBank under the accession numbers CP062229 to CP062230 and CP062231 to CP062234, respectively. Sequences of single genes are available at NCBI GenBank under the accession numbers MW915806 to MW915808 and MW917139 to MW917144.

## Figures and Tables

**FIG 5:**
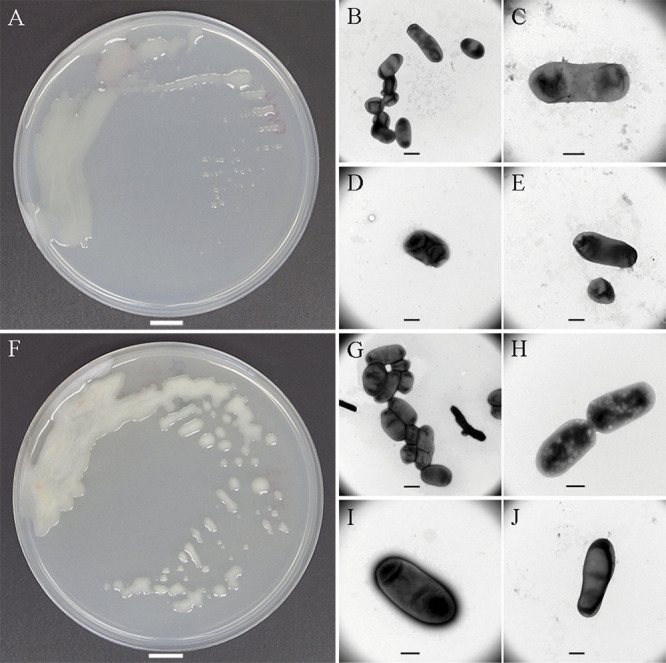
Colonies and cells of Mesorhizobium onobrychidis strain OM4^T^ and Onobrychidicola muellerharveyae strain TH2^T^. (A) Colonies of OM4^T^ on YMA after 10 days incubation at 28°C. (B to E) Transmission electron microscopy (TEM) micrographs of cells of OM4^T^ grown in YMA. (F) Colonies of O. muellerharveyae strain TH2^T^ on YMA after 10 days incubation. (G to J) TEM micrographs of cells of O. muellerharveyae strain TH2^T^ grown in YMA liquid. Scale bar, 100 mm in panels A and F, 1 μm in panels B and G, and 500 nm in panels C, D, E, H, and I.

## APPENDIX

### Formal descriptions of two new *Rhizobiales* species, including a new genus in *Rhizobiaceae*.

The following paragraphs provide formal descriptions (protologues) of a new *Rhizobiaceae* genus and two new *Rhizobiales* species (Fig. 5).

### Description of Mesorhizobium onobrychidis sp. nov.

Mesorhizobium onobrychidis (o.no.bry’chi.dis; N.L. gen. n. *onobrychidis* of the plant genus *Onobrychis*).

Cells are Gram-negative, non-spore forming, and rod-shaped, 1.1 to 2.3 μm (1.7 ± 0.3 standard deviation [SD]) in length, and 0.7 to 1.15 μm (0.95 ± 0.1 SD) wide (*n* = 15). They are nonmotile and nonflagellated, aerobic, and oxidase and catalase positive. Bacteria grow on YMA, tryptone-yeast extract (TY), and R2A media. Colonies are very slow-growing on YMA medium, appearing within 8 to 10 days, and are white, glistening, circular, and convex with 1 mm diameter after 10 days incubation at 28°C. Growth is observed at temperatures between 10 and 25°C. Nitrate reduction is negative. Glucose fermentation is negative. Arginine dihydrolase and β-galactosidase tests are negative. Production of urease is positive. Glucose, l-arabinose, d-mannose, d-mannitol, d-maltose, gluconate, and malate are assimilated. A weak assimilation was observed for caprate. Adipate, citrate, and phenylacetate are not assimilated. The major fatty acids (>5%) are C_18:1_ ω7c, C_16:0_, C_19:0_ cyclo ω7c, 11-methyl C_18:1_ ω7c, and C_18:0_. OM4^T^ induces effective nodules on its original host plant (cultivars Taja, Perly, and ONO 20). Additionally, genes involved in legume nodulation and nitrogen fixation were identified in the genome of the strain OM4^T^.

The genome size of the type strain (OM4^T^) is 7.55 Mb. The genome is composed of the circular chromosome (7.32 Mb) and circular plasmid (227 kb). The G+C content of total genomic and/or chromosomal DNA is 61.9%.

The type strain OM4^T^ (DSM 109849, NCCB 100791) was isolated from a root nodule of Onobrychis viciifolia, Germany, in 2019. The DDBJ/EMBL/GenBank accession numbers for the genome sequence are CP062229 (chromosome) and CP062230 (plasmid).

### Description of *Onobrychidicola* gen. nov.

*Onobrychidicola* (O.no.bry.chi.di’co.la; N.L. fem. n. *Onobrychis*, a plant genus; L. suff. -*cola* [from L. masc. or fem. n. *incola*], inhabitant, dweller; N.L. masc. n. *Onobrychidicola*, a dweller of *Onobrychis*).

Cells are aerobic, Gram negative, non-spore forming, rod-shaped, nonmotile, and nonflagellated. They are oxidase and catalase positive. The major fatty acids (>5%) are C_18:1_ ω7c, C_19:0_ cyclo ω7c, and C_16:0_ and C_17:0_ cyclo ω7c. The G+C contents of total genomic and chromosomal DNA of the type strain of the type species are 60.4 and 60.6%, respectively. The genus *Onobrychidicola* has been separated from other *Rhizobiaceae* genera based on core genome phylogeny, whole- and core proteome comparisons (wpAAI and cpAAI, respectively), as well as pan-genome and functional analyses.

The type species is Onobrychidicola muellerharveyae.

### Description of Onobrychidicola muellerharveyae sp. nov.

Onobrychidicola muellerharveyae (muel.ler.har’vey.ae; N.L. gen. n. muellerharveyae, named in honor of Irene Mueller-Harvey for her outstanding work on sainfoin).

Cells are aerobic, Gram negative, non-spore forming, rod-shaped, 1.44 to 2.63 μm (2.0 ± 0.3) in length, 0.8 to 1.4 μm (1.1 ± 0.1) wide (*n* = 20), nonmotile, and nonflagellated. It is an oxidase- and catalase-positive bacterium that shows relatively good growth on YMA, TY, and R2A media. Colonies are slow-growing on YMA medium, whitish to pale creamy, variable in size and shape, and 1 to 4 mm diameter after 10 days growth at 28°C. Growth was observed at a temperature range between 10 and 30°C. Nitrate reduction and glucose fermentation are negative. Arginine dihydrolase and gelatin hydrolysis tests are negative. Production of urease and esculin hydrolysis is positive. Production of β-galactosidase is weakly positive. d-Mannose and d-glucose are assimilated. A weak assimilation was observed for l-arabinose, d-mannitol, d-maltose, gluconate, and caprate. Adipate, malate, citrate, and phenylacetate are not assimilated. The major fatty acids (>5%) are C_18:1_ ω7c, C_19:0_ cyclo ω7c, and C_16:0_ and C_17:0_ cyclo ω7c. The strain TH2^T^ could not induce nodules on its original host plant. Accordingly, genes involved in legume nodulation and nitrogen fixation were absent in the genome of strain TH2^T^.

The genome size of the type strain (TH2^T^) is 6.44 Mb. The genome is composed of the circular chromosome (5.88 Mb) and three circular plasmids (98 to 238 kb). The G+C content of total genomic and chromosomal DNA is 60.4 and 60.6%, respectively.

The type strain TH2^T^ (DSM 109848^T^, NCCB 100790^T^) was isolated from a root nodule of Onobrychis viciifolia in Germany in 2019. The DDBJ/EMBL/GenBank accession numbers for the genome sequence are CP062231 to CP062234.
